# Highly efficient heat-dissipation power driven by ferromagnetic resonance in *M*Fe_2_O_4_ (*M* = Fe, Mn, Ni) ferrite nanoparticles

**DOI:** 10.1038/s41598-022-09159-z

**Published:** 2022-03-28

**Authors:** Jae-Hyeok Lee, Yongsub Kim, Sang-Koog Kim

**Affiliations:** National Creative Research Initiative Center for Spin Dynamics and Spin-Wave Devices, Nanospinics Laboratory, Research Institute of Advanced Materials, Department of Materials Science and Engineering, Seoul National University, Seoul, 151-744 South Korea

**Keywords:** Ferromagnetism, Magnetic properties and materials

## Abstract

We experimentally demonstrated that heat-dissipation power driven by ferromagnetic resonance (FMR) in superparamagnetic nanoparticles of ferrimagnetic *M*Fe_2_O_4_ (*M* = Fe, Mn, Ni) gives rise to highly localized incrementation of targeted temperatures. The power generated thereby is extremely high: two orders of magnitude higher than that of the conventional Néel-Brownian model. From micromagnetic simulation and analytical derivation, we found robust correlations between the temperature increment and the intrinsic material parameters of the damping constant as well as the saturation magnetizations of the nanoparticles’ constituent materials. Furthermore, the magnetization–dissipation-driven temperature increments were reliably manipulated by extremely low strengths of applied AC magnetic fields under resonance field conditions. Our experimental results and theoretical formulations provide for a better understanding of the effect of FMR on the efficiency of heat generation as well as straightforward guidance for the design of advanced materials for control of highly localized incrementation of targeted temperatures using magnetic particles in, for example, magnetic hyperthermia bio-applications.

## Introduction

**Figure 1:**
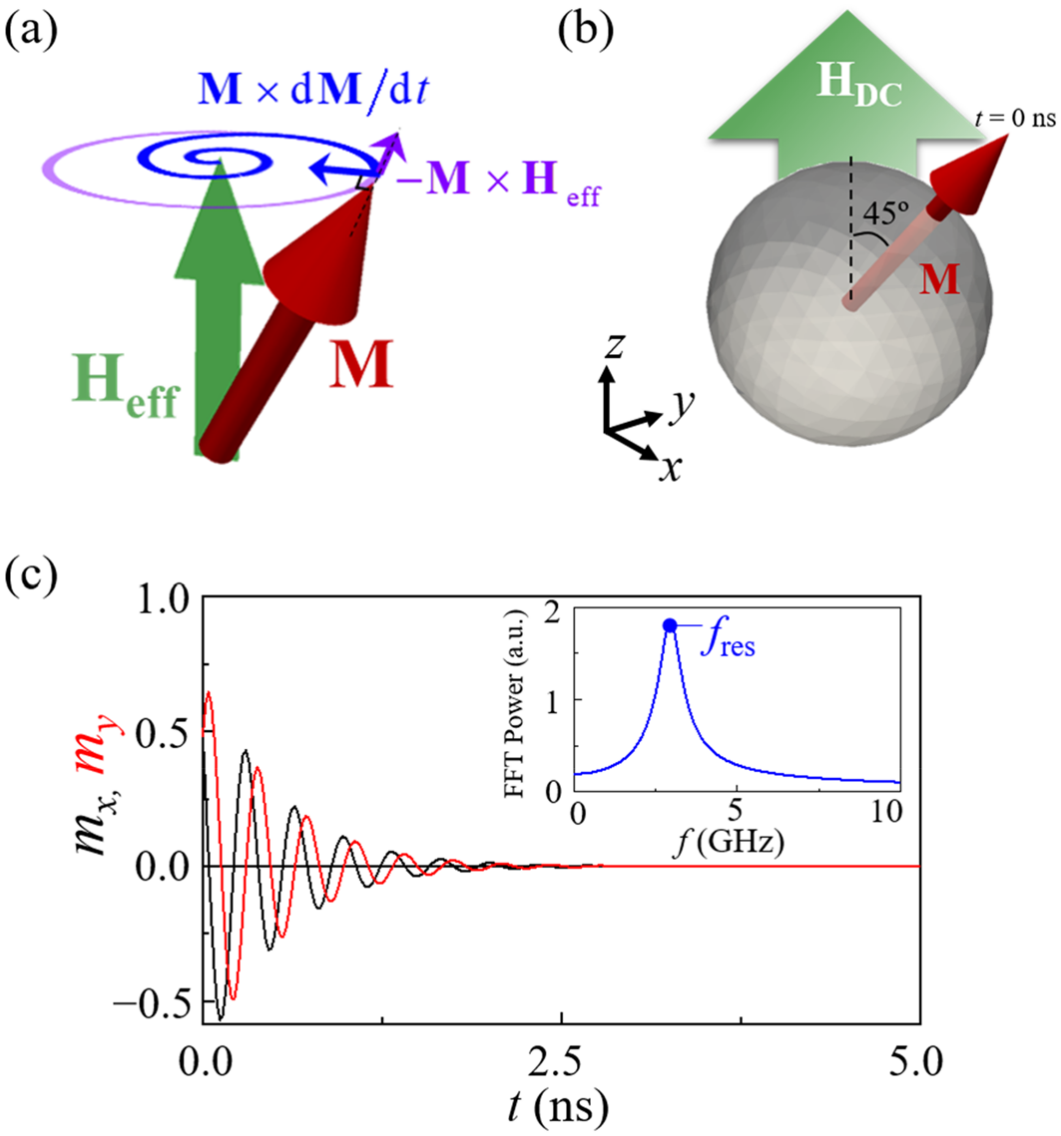
(**a**) Schematic illustration of two different precession motions of magnetization vector **M** (red) around effective magnetic field vector **H**_eff_ (green) with (blue) and without (purple) damping. (**b**) Single nanosphere of diameter 2*R* = 12 nm and MnFe_2_O_4_ with overall magnetizations’ orientation being deviated 45° from direction (+ z) of DC magnetic field **H**_DC_. (**c**) Micromagnetic simulations of the temporal oscillations of *m*_*x*_ and *m*_*y*_ components of **M** during relaxation process under *H*_DC_ = 1010 Oe for a 12 nm MnFe_2_O_4_ nanosphere. The inset shows the FFT power spectrum of the *m*_*x*_ oscillation, wherein the peak position corresponds to the intrinsic precession frequency at the given strength of *H*_DC_.

Ferromagnetic resonance (FMR) is a longstanding well-known phenomenon in the research field of magnetism, and is one of the fundamental dynamic modes associated with the precession of individual magnetizations at an intrinsic resonance frequency. Once the precession motion of magnetizations **M** occurs around a given direction of effective magnetic field **H**_eff_, it typically encounters resistance owing to intrinsic damping, consequently resulting in decreasing amplitude of the in-plane component of **M** perpendicular to **H**_eff_ in order to allow the orientation of **M** in the direction of **H**_eff_ (see Fig. 1a). Such precession motions have been well described by the Landau-Lifshitz-Gilbert equation, $d{\mathbf{M}}/dt{\text{ }} =  - \gamma {\mathbf{M}} \times {\mathbf{H}}_{{{\text{eff}}}}  + (\alpha /M_{{\text{S}}} ){\mathbf{M}} \times d{\mathbf{M}}/dt$ with *α* the dimensionless Gilbert damping constant, *γ* the gyromagnetic ratio, and *M*_S_ the saturation magnetization of a given material. For example, when the orientations of individual magnetizations inside a model sphere are deviated from the direction of **H**_DC_ applied to the sphere (see Fig. 1b), the magnetization **M** precesses about **H**_DC_ with the decreasing amplitude of the *m*_*x*_ and *m*_*y*_ oscillations, as shown in Fig. 1c. The fast Fourier transformation (FFT) of the temporal oscillations of the *m*_*x*_ and *m*_*y*_ components determine an intrinsic precession frequency, *f*_res_, as shown in the inset of Fig. 1c.

Since the FMR effect was experimentally discovered in the 1940s^1,2^, FMR measurement techniques have been used to probe magnetization excitations in many magnetic systems including magnetic thin films^3^ and magnetic dots of different shapes such as finite rectangular elements^4,5^, circular nanodots^6^, and magnetic nanowires^7^. Magnetization excitation and the attendant relaxation processes in finite-dimension magnetic systems result in a variety of dynamic motions such as magnetic domain-wall motions^8,9^, many spin-wave modes^10,11^, and novel dynamic motions of magnetic vortices^12^ and skyrmions^13^. Furthermore, research interest in the magneto-thermal effect, which represents the conversion of magnetostatic energy to heat due to intrinsic damping, recently has grown. When microwave magnetic fields are applied to excite magnetizations in a magnet, the field energy can be converted to one or another energy form (e.g., heat), thus leading to temperature increments during the processes of magnetization excitation and dissipation. Therefore, the heat-dissipation mechanism is promising for potential applications to spintronics and magnonics^14–19^ as well as magnetic hyperthermia bio-applications^20,21^.

The microscopic origin of damping has been intensively studied^22–24^ in the research area of magnetization dynamics; however, direct experimental measurements of FMR-driven heat generation have been reported only in several studies on thermoelectric detection^25,26^, the bolometric effect^18,27^, and mechanical detection via the magnetostriction effect^14^. In our earlier theoretical and numerical calculation work in Ref.^28^, we proposed an idea on how to obtain ultra-high heating power by deriving explicit forms of the energy-dissipation rate (heating power) in Permalloy nanospheres under specific resonance conditions. In Ref.^20^, we also experimentally demonstrated that high-efficiency heat generation can be achieved through resonant spin-excitation and dissipation mechanism.

In the present study, by the combination of analytical derivation, micromagnetic simulation, and experimental verification, we explored temperature incrementation through local heating under FMR in superparamagnetic nanoparticles of three different ferrimagnetic materials, namely Fe_3_O_4_, MnFe_2_O_4_, and NiFe_2_O_4_. The measured temperature increments were associated directly with FMR-driven heat-dissipation power that is two orders of magnitude greater than that driven by Néel-Brown relaxation mechanisms^29^. We further correlated the temperature increments with the saturation-magnetization and damping-constant parameters of three different constituent materials, as well as the parameters of the strengths of AC and DC fields and of AC field frequency. No experimental correlations between FMR driven heat-dissipation power and temperature increment have yet been reported in terms of material parameters or low strengths of AC field under resonance field conditions, except for our earlier theoretical study^28^. This work offers necessary guidance for design of advanced materials that can be utilized for generation of efficient heat dissipation and control of the increment of targeted temperature in a highly local area.

## Results and discussion

### Energy-dissipation rate *Q*

**Figure 2:**
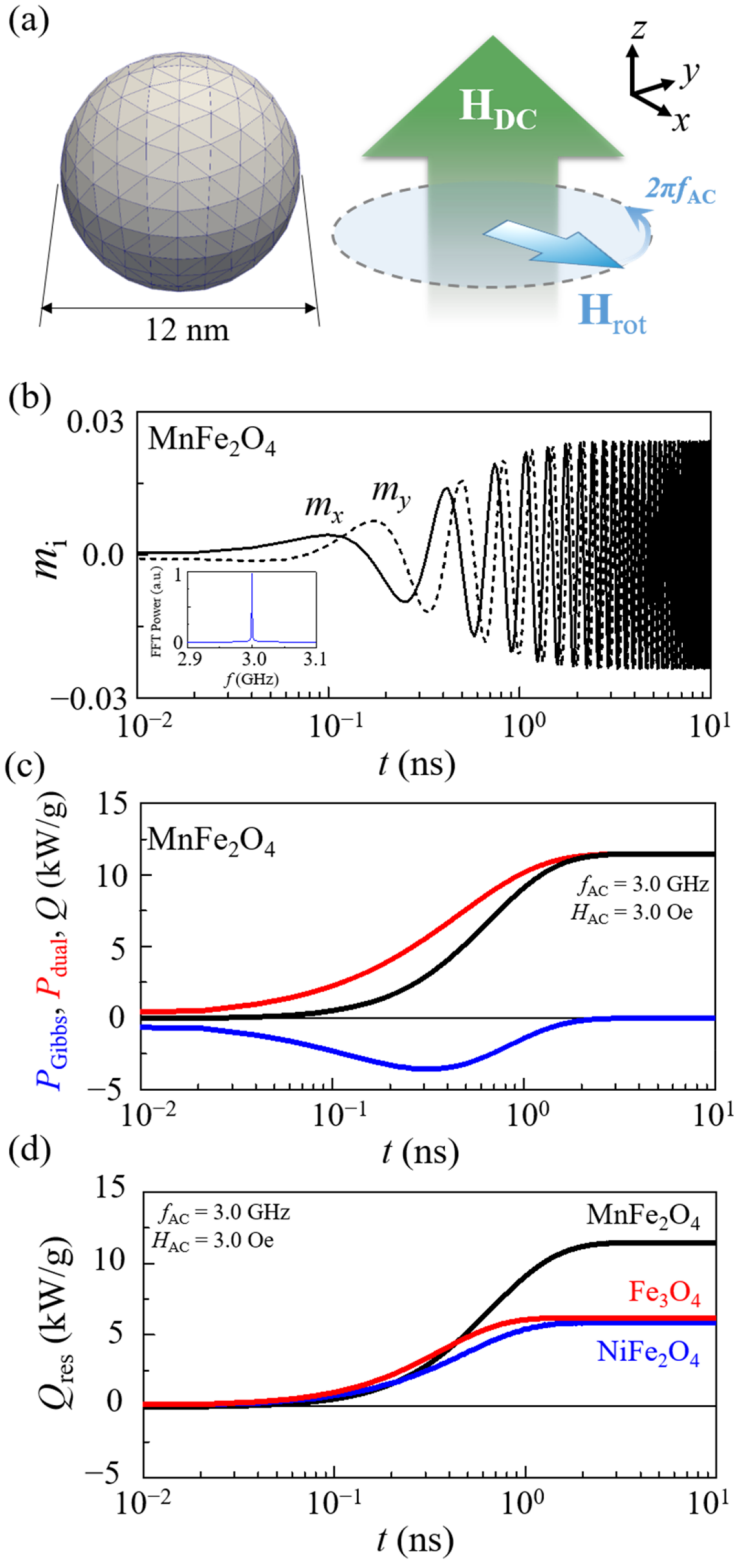
(**a**) Single-domain nanosphere of diameter 2*R* = 12 nm and MnFe_2_O_4_ under application of *H*_DC_ = 1010 Oe in + z direction. (**b**) Upon application of CCW rotating field (*f*_AC_ = 3.0 GHz, *H*_AC_ = 3.0 Oe) on *xy* plane, *x* and *y* components of magnetization start to oscillate with increasing amplitude with time, and then reach steady state of precession at specific polar angle of ~ 2º. The inset shows the FFT power, which indicates that the oscillation frequency is the same as *f*_AC_ = 3.0 GHz. (**c**) Temporal variation of *P*_Gibbs_ (blue line) and *P*_dual_ (red line), *Q* (black line) = *P*_Gibbs_ + *P*_dual_, calculated using simulation data under resonance field condition (*f*_AC_ = 3.0 GHz and *H*_DC_ = 1010 Oe) with *H*_AC_ = 3 Oe. (d) $\bar{Q}_{{{\text{res}}}}$ variation for MnFe_2_O_4_ (black line), Fe_3_O_4_ (red line), and NiFe_2_O_4_ (blue line) for same frequency of *f*_AC_ = 3.0 GHz with *H*_AC_ = 3 Oe, but with different DC field strengths *H*_DC_ = 970, 1010, and 980 Oe, respectively, to meet corresponding resonance conditions.

In order to numerically estimate heat-dissipation power (the energy-dissipation rate) during magnetization excitation and relaxation processes in magnetic particles, we first conducted a micromagnetic simulation of a model sphere of 12 nm diameter (see left of Fig. 2a) and composed of ferrimagnetic MnFe_2_O_4_ (for details, see Supplementary Material S1). The model includes magnetizations oriented in the direction of an applied DC field in the initial state. Then, to excite the magnetizations with a low field strength, we choose a counter clock-wise (CCW) rotating field ${\mathbf{H}}_{{{\text{rot}}}} = H_{{{\text{AC}}}} \cos (2\pi f_{{{\text{AC}}}} t){\hat{\mathbf{x}}} + H_{{{\text{AC}}}} \sin (2\pi f_{{{\text{AC}}}} t){\hat{\mathbf{y}}}$ on the *xy* plane, as illustrated in the right of Fig. 2a, because this field is the resonant eigen-basis of the CCW precession motion of **M**. We should note that CW rotating fields lead to no precession motion of **M**. Upon application of **H**_rot_ with *f*_AC_ = *f*_res_ = 3.0 GHz and *H*_AC_ = 3 Oe, the precession starts and thus the *m*_*x*_ and *m*_*y*_ components oscillate periodically with increasing amplitude with time under the resonance condition (*f*_res_ = 3.0 GHz and *H*_DC_ = 1010 Oe), as shown in Fig. 2b. At the given field strength of *H*_AC_, the *m*_*x*_ and *m*_*y*_ oscillatory amplitudes reach the steady state with a certain polar angle of ~ 2° and then precess sustainably at that angle until the rotating field is sustained.

According to the inherent and irreversible nature of the magnetization dynamic process, the field energy injected into the sphere model is transformed into heat through magnetization dissipation. In order to quantify the amount of heat-dissipation power during the continuous precession/relaxation dynamics in the model sphere, we numerically calculated the magnetic energy-dissipation rate (power loss) *Q* from the simulation data shown in Fig. 2b on the basis of energy conservation and fundamental Maxwell equations. The time-varying external magnetic forces constitutes dual power, which contributes the time variation of Gibbs free energy as well as energy dissipation rate^30^. Therefore, the resultant power loss can be given as the sum of Gibbs free energy density $P_{{{\text{Gibbs}}}}  =  - \left[ {1/\left( {\rho V} \right)} \right]\int_{V} {\left( {{\text{d}}\varepsilon _{{{\text{Gibbs}}}} /{\text{d}}t} \right)}  dV$ and the dual power density $P_{{{\text{dual}}}}  =  - \left[ {1/\left( {\rho V} \right)} \right]\int_{V} {\left( {{\mathbf{M}} \cdot {\text{d}}{\mathbf{H}}_{{{\text{ext}}}} /{\text{d}}t} \right)}   dV$, where $\varepsilon_{{{\text{Gibbs}}}}$ is the Gibbs free energy density, and *V* and *ρ* are the volume and the density of magnetic material, respectively. Figure 2c compares the resultant calculations of *P*_Gibbs_, *P*_dual_, and *Q* (= *P*_Gibbs_ + *P*_dual_) for MnFe_2_O_4_ under the resonance field condition (*f*_AC_ = *f*_res_ = 3.0 GHz at *H*_DC_ = 1010 Oe) with *H*_AC_ = 3.0 Oe. *P*_dual_ (red) gradually increases and then reaches a certain constant value, while *P*_Gibbs_ (blue) decreases in the beginning and then converges to zero in the steady state. Thus *Q* turns out to be equal to *P*_dual_ at the steady state. Accordingly, the steady-state *Q* values at resonance (noted as $\bar{Q}_{{{\text{res}}}}$) could be obtained using $P_{{{\text{dual}}}}  =  - \left[ {1/\left( {\rho V} \right)} \right]\int_{V} {\left( {{\mathbf{M}} \cdot {\text{d}}{\mathbf{H}}_{{{\text{ext}}}} /{\text{d}}t} \right)} dV$ from the simulation data, as illustrated in Fig. 2c. Figure 2d compares $Q_{{{\text{res}}}}$ for the three different materials Fe_3_O_4_, MnFe_2_O_4_, and NiFe_2_O_4_, and we finally obtained the steady-state $Q_{{{\text{res}}}}$ values, $\bar{Q}_{{{\text{res}}}}$ = 6.2, 11.5, and 5.8 kW/g, respectively. Surprisingly, these estimated values are one or two orders of magnitude greater than the typical values (0.1–1 kW/g) of specific loss power (SLP) obtained from Fe_3_O_4_^31,32^_,_ Fe_2_O_3_^33^, etc., by conventional means, which represents the initial rate of release of heat from a unit weight of magnetic material during exposure to an oscillating magnetic field according to the conventional Néel-Brown relaxation mechanism^29^. Such large energy dissipation rates are very promising with respect to the efforts to achieve efficient, fast heat generation using magnetic nanoparticles.

### Measurements of temperature increments in ***M***Fe_2_O_4_ (***M*** = Fe, Mn, Ni) nanoparticles

**Figure 3:**
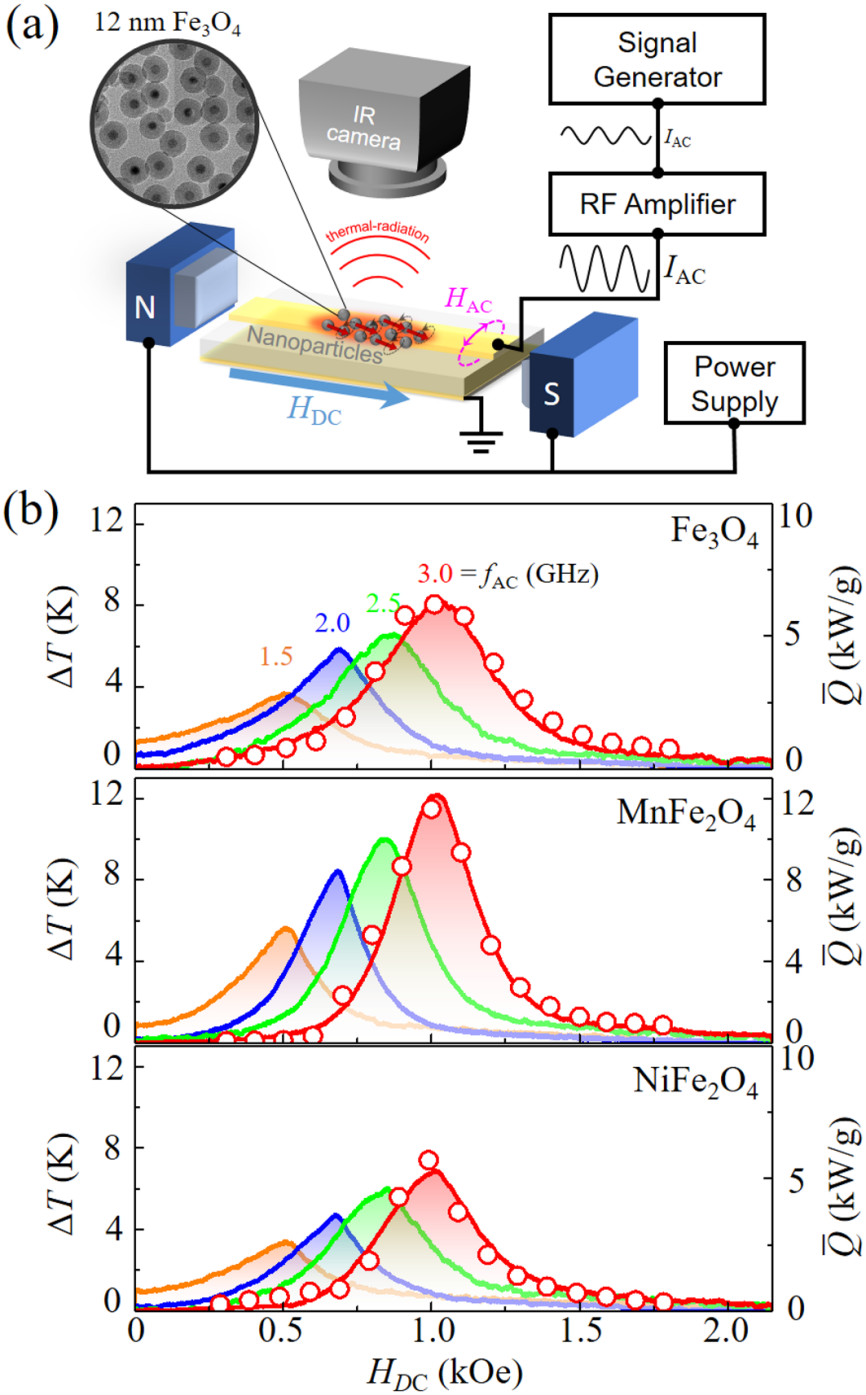
(**a**) Schematic illustration of experimental setup for measurements of temperature incrementation directly from nanoparticles by thermal radiation using IR camera during FMR excitations. To generate AC magnetic fields, radio-frequency (RF) currents of GHz frequencies were transmitted to the microstrip using a signal generator and an RF amplifier. *H*_DC_ was applied on the axis of a microstrip line so that the direction of **H**_AC_ could be perpendicular to the direction of **H**_DC_. (**b**) Temperature-increment spectra measured with increasing *H*_DC_ strength (at constant rate of 7 Oe/s) for *M*Fe_2_O_4_ (*M* = Mn, Fe, Ni) nanoparticles for each of *f*_AC_ = 1.5, 2.0, 2.5, and 3.0 GHz with the same value of *H*_AC_ = 3.0 Oe. The open circles on the *f*_AC_ = 3.0 GHz spectrum indicate steady-state heat-dissipation rate $\bar{Q}$ calculated from micromagnetic simulation data under the same resonant conditions as those for the experimental measurement of the ∆*T* spectra.

Based on the above calculation of heat-dissipation power in nanoparticles, we set up the apparatus schematically illustrated in Fig. 3a in order to experimentally verify the temperature increments by heat dissipation from nanoparticles without any environmental aqueous solutions. The apparatus is composed mainly of two separate parts: a microwave power pumping system to allow for magnetization excitations, and a temperature probing system to detect thermal radiation from the particles (for details, see Supplementary Materials S2–S5). Temperature increments were measured directly from the magnetic nanoparticles covered with silica shells of 12 nm thickness to avoid their agglomeration, as shown in the inset of Fig. 3a. The purpose of the silica-shell coating around each magnetic particle is to suppress inter-dipolar and inter-exchange interactions between the individual particles, thus allowing for reliable measurements of heat-dissipation power from ensemble-averaged isolated particles (see Supplementary Materials S3 and S4 for further information). To compare the quantitative values of the temperature increments from the nanoparticles of the three different materials Fe_3_O_4_, MnFe_2_O_4_, and NiFe_2_O_4_, in Fig. 3b we plotted the ∆*T* spectra as measured by increasing DC field strength in the range of *H*_DC_ = 0 ~ 2 kOe (at a rate of 7 Oe/s) for each of the different frequencies of *f*_AC_ = 1.5, 2.0, 2.5, and 3.0 GHz with a single constant value of *H*_AC_ = 3.0 Oe. The ∆*T*-vs-*H*_DC_ spectra resemble characteristic FMR spectra with single peaks maximized at certain *H*_DC_ values according to $f_{{{\text{res}}}}  = \left( {\gamma /2\pi {\text{ }}} \right)(H_{{{\text{DC}}}}  + H_{{{\text{int}}}} )$ with *H*_int_ internal fields including a magnetocrystalline anisotropy field and intra-dipolar interaction inside each magnetic particle. Note that, since the DC field strength (~ 1 kOe) is sufficiently higher than the internal field (~ 0.15 kOe) owing to the randomly oriented anisotropy axes of individual particles, the precession frequency and its related heating power are affected dominantly by the applied DC field strength. The height and position of the maximum peak in each spectrum are remarkably varied with *f*_AC_. On the other hand, the peak position does not much change with the constituent material, while the peak height rather varies with the material. The slight changes (~ 30 Oe for *f*_AC_ = 3.0 GHz) in the DC field position of the maximum peak for the different constituent materials are related to the insignificant difference in the Gilbert gyromagnetic ratio *γ*_G_ (= 2.712, 2.764, and 2.751 MHz/Oe) as well as in the internal field *H*_int_ (= 174.4, 124.8, and 160.7 Oe) for Fe_3_O_4_, MnFe_2_O_4_ and NiFe_2_O_4_, respectively. These values were obtained from FMR measurements of the samples (for further details, see Supplementary Materials S3 and S4).

The ∆*T*-vs-*H*_DC_ spectra for Fe_3_O_4_, shown in the top panel of Fig. 3b, exhibit different peak positions at *H*_DC_ = 502, 685, 862, and 1030 Oe for *f*_AC_ = 1.5, 2.0, 2.5, and 3.0 GHz, respectively. On the other hand, the peak height increases with *f*_AC_ such that the maximum temperature increments were found to be ∆*T*_max_ = 3.7, 5.9, 6.6, and 8.3 K for *f*_AC_ = 1.5, 2.0, 2.5, and 3.0 GHz, respectively. These behaviors are associated with the FMR power spectra given by the Kittel equation for a spherical model in single-domain state^2^ $f_{{{\text{res}}}}  = \left( {\gamma /2\pi {\text{ }}} \right)H_{{{\text{eff}}}}$. For the other two samples of MnFe_2_O_4_ and NiFe_2_O_4_, the same trends were observed, but the peak heights were found to vary with the constituent material. For example, for *f*_AC_ = 3.0 GHz, Δ*T*_max_ = 12.2 (at *H*_DC_ = 1014 Oe) and 6.9 K (at *H*_DC_ = 1017 Oe) were found for MnFe_2_O_4_ and NiFe_2_O_4_, respectively.

### Comparison of energy-dissipation rate *Q* and temperature increments Δ*T*

According to the differential equation of Newton’s law of heating (cooling), the quantity of the energy-dissipation rate *Q* can be associated with Δ*T*, which temperature increment, as driven by FMR, converges to a saturation value within just a few seconds^20^. Therefore, in order to understand such temperature-incremental behaviors and their underlying mechanism as well as the material dependence of Δ*T*_max_*,* we accordingly compared the numerical calculations of the steady-state energy-dissipation rate $\bar{Q}$ as a function of *H*_DC_ in the vicinity of the resonance peaks (see open circles). Since *P*_Gibbs_ always converges to zero in the steady states, as previously shown in Fig. 2c, $\bar{Q}$ can be obtained by directly calculating the dual power density, $P_{{{\text{dual}}}}  =  - [1/(\rho V)]\int_{V} {({\mathbf{M}} \cdot {\text{d}}{\mathbf{H}}_{{{\text{ext}}}} /{\text{d}}t)\;} dV$ from the simulation data of the three different materials under the field conditions of *f*_AC_ = 3 GHz and *H*_AC_ = 3.0 Oe, as examples. The simulation data were compared with the temperature increments measured from the real samples only for the case of *f*_AC_ = 3.0 GHz, where a sufficiently large value of *H*_DC_ (~ 1 kOe) relative to *H*_int_ had been applied; thus, most of the magnetizations were aligned in the direction of *H*_DC._ The data points (open symbols) of the $\bar{Q}$-vs-*H*_DC_ spectra are in excellent agreement with the measured temperature increments (red solid curves), confirming that the temperature increments originate specifically from the heat (energy) dissipation driven by the FMR excitation/relaxation processes. Since the quantity of $\bar{Q}$ can be readily manipulated by tuning *H*_DC_ as well as the AC field frequency, targeting temperatures also can be manipulated by those field parameters.

Furthermore, the steady-state energy-dissipation rate $\bar{Q}$ was found to have its maximum peak value at resonance. This maximum value, denoted as $\bar{Q}_{{{\text{res}}}}$, can be analytically expressed for *H*_rot_ basis as^28^

1 $$  \bar{Q}_{{{\text{res}}}}  = (1/\alpha )(\gamma M_{S} H_{{{\text{AC}}}}^{2} /\rho )  $$

with *ρ* the density of magnetic material for the constraint *H*_AC_ < *αH*_DC_. For application of linear AC fields, $\bar{Q}_{{{\text{res}}}}$ is just one-fourth of that for application of the corresponding CCW rotating field, since the CW rotating field does not contribute to the precession, and also because the field strength of the CCW rotating field is just half of the field strength of a linear oscillating field (for further understanding, see Supplementary Material S1). Our experimental field conditions meet the constraints of *H*_AC_ < *αH*_DC_ such as *H*_AC_ < 5 Oe, *αH*_DC_ = 117 Oe with $H_{{{\text{DC}}}} = 1030\,{\text{Oe}}$ and *α* = 0.114 for MnFe_2_O_4_ at *f*_res_ = 3.0 GHz. Therefore, we can use analytical Eq. (1) to correlate the material parameters with the experimental data of Δ*T*_max_. Since $\bar{Q}_{{{\text{res}}}}$ is proportional to 1/*α* and *M*_S_, we can directly compare Δ*T*_max_ for the three different materials of *M*Fe_2_O_4_ (*M* = Fe, Mn, Ni) at the same resonance frequency *f*_res_ = 3.0 GHz; the $\bar{Q}_{{{\text{res}}}}$ from Eq. (1) are 11.6 kW/g for MnFe_2_O_4_ [*M*_1kOe_ = 85.5 emu/g (See Supplementary information S2 for additional details regarding structual, magnetic propreties and MS normalization] and *α* = 0.114), 7.8 kW/g for Fe_3_O_4_ (*M*_1kOe_ = 95.9 emu/g and *α* = 0.180), and 6.5 kW/g for NiFe_2_O_4_ (*M*_1kOe_ = 81 emu/g and *α* = 0.134). These values as determined from the analytical form agree quantitatively well with the micromagnetic simulation data discussed earlier in Fig. 2b, and with the experimentally measured Δ*T*_max_ for the three samples. Thus, Eq. (1) can be considered to be very useful for design of appropriate nanoparticle material by choosing intrinsic material parameters of *α* and *M*_S_.

### $\bar{\text{Q}}$_res_ and Δ***T***_max_ dependence upon ***H***_AC_

**Figure 4:**
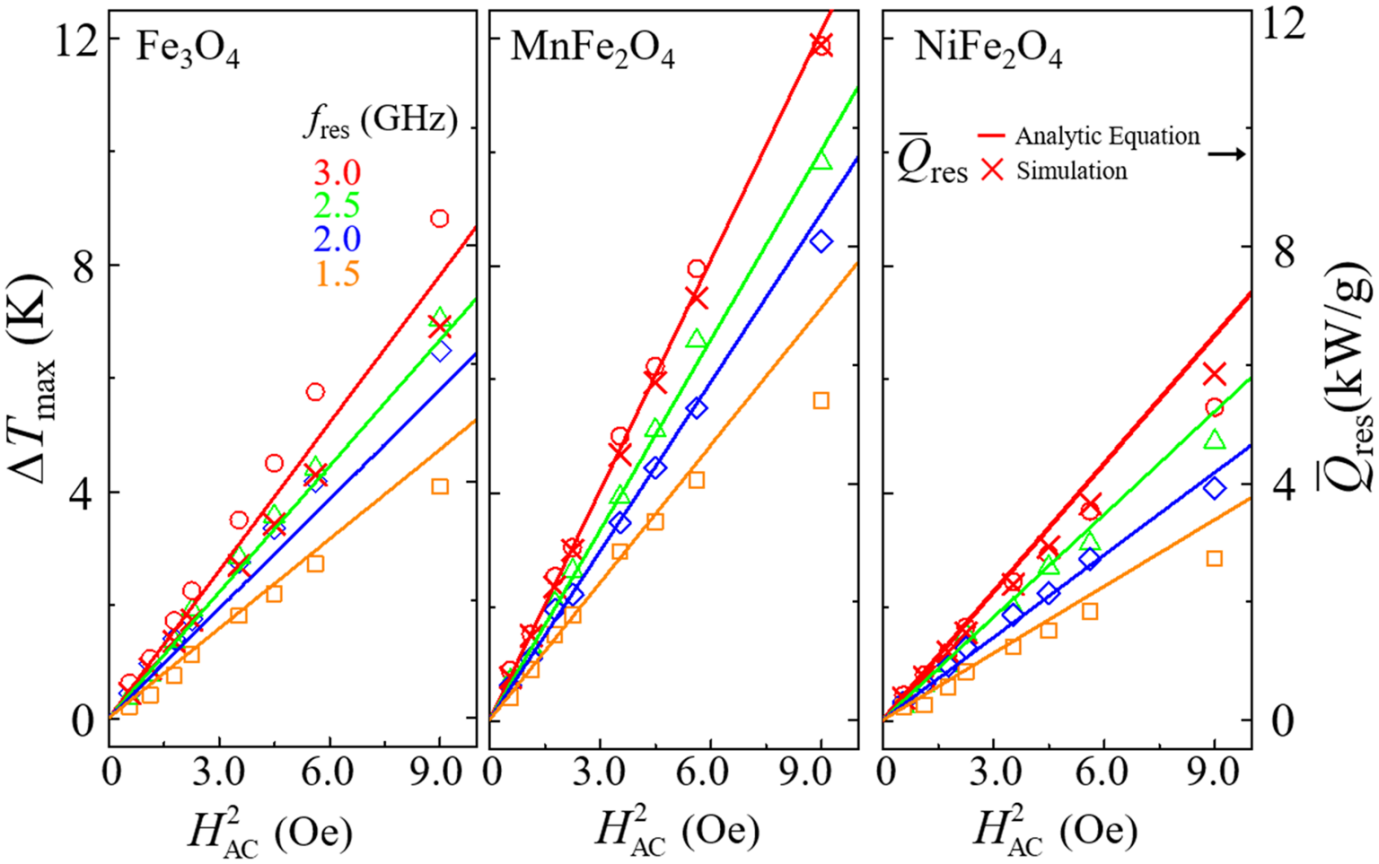
Plots of maximum temperature increments ∆*T*_max_ as function of $H_{{{\text{AC}}}}^{2}$ for Fe_3_O_4_ (left panel), MnFe_2_O_4_ (middle panel), and NiFe_2_O_4_ (right panel) materials, as obtained at resonance with each of different frequencies of *f*_AC_ = 1.5, 2.0, 2.5, and 3.0 GHz. The red solid lines and the cross symbols indicate the steady-state energy-dissipation rate at resonance $\bar{Q}_{{{\text{res}}}}$ obtained from the analytic equation [Eq. (1)] and the simulation data for *f*_AC_ = *f*_res_ = 3.0 GHz, respectively.

The analytical form of $\bar{Q}_{{{\text{res}}}}$ also shows only the $H_{{{\text{AC}}}}^{{}}$ dependence, because the *H*_DC_ / *f*_AC_ relation is constrained by the resonance field condition. To experimentally verify the $\bar{Q}_{{{\text{res}}}}$ dependence upon $H_{{{\text{AC}}}}^{{}}$, we further measured Δ*T*_max_ as a function of *H*_AC_ for the three different samples for each field frequency of *f*_AC_ = 1.5, 2.0, 2.5, and 3.0 GHz (see open symbols in Fig. 4). To verify the relation of $\bar{Q}_{{{\text{res}}}}$ to Δ*T*_max_, we plotted analytical calculations (solid lines) of $\bar{Q}_{{{\text{res}}}}$ and corresponding simulation data (cross symbols). For illustrative simplicity, the micromagnetic simulation data represent only the case of *f*_AC_ = *f*_res_ = 3.0 GHz and *H*_AC_ = 3.0 Oe. The overall data on Δ*T*_max_ and $\bar{\text{Q}}$_res_, shown in Fig. 4, are in agreement in terms of $H_{{{\text{AC}}}}^{2}$ dependence.

For MnFe_2_O_4_ (middle panel), Δ*T*_max_ and $\bar{\text{Q}}$_res_ agree quite well except for *f*_AC_ = 1.5 GHz. This slight difference between the experimental data on Δ*T*_max_ and the analytical and simulation data on $\bar{\text{Q}}$_res_ was due to the fact that the DC field strength was not sufficient to saturate magnetizations in the real samples under the resonance condition of *f*_AC_ = 1.5 GHz. On the other hand, for Fe_3_O_4_ and NiFe_2_O_4_, relatively large differences between Δ*T*_max_ and $\bar{\text{Q}}$_res_ are likely that we could not include the exactly same value of the internal field of each sample, being caused by the magnetocrystalline anisotropy field and intra-dipolar field arising from disorders of the surface and volume of each particle. The above results clearly indicate that a highly efficient energy-dissipation rate driven by FMR directly leads to temperature increments from nanoparticles, depending on the given intrinsic material parameters. Also, the temperature increments are reliably controllable by adjusting the parameters of the DC and AC field strengths and the AC field frequency.

## Summary

We demonstrated a considerable magneto-thermal effect driven by resonant magnetization precession/relaxation in superparamagnetic nanoparticles of ferrimagnetic oxides. The extra-ordinarily high energy-dissipation rate (power loss) during resonance magnetization dynamics was evidenced by experimental measurements of temperature increments in the nanoparticles, with the help of analytical calculation as well as micromagnetic simulations. In comparison with other mechanisms related to power loss, such as the Néel-Brownian model, the amount of heat dissipation can be significantly enhanced, via resonant spin-excitation and relaxation, by about two orders of magnitude larger than by other means. We experimentally explored robust correlations between the temperature increment and the intrinsic material parameters: i.e., the damping constant as well as the saturation magnetizations of three different constituent materials, *M*Fe_2_O_4_ (*M* = Fe, Mn, Ni). It revealed that the heat-dissipation power is proportional to the saturation magnetization and inversely proportional to the damping parameter of a constituent material. In order to further maximize the power loss, such critical magnetic parameters can be tailored optimally through the existing novel engineering techniques: saturation magnetization can be increased by substitution of transition metal ions in iron-oxides^34^, synthesis of bimagnetic core–shell materials^35^, or the thermolysis process^36^. Also, Gilbert damping constants can be reduced by the annealing process^37^, the optimal stoichiometric composition^38,39^, the strain-engineered process^40^, or other means. The measured temperature increments were also well controllable with low-magnetic-field-strength parameters. The present work quantitatively clarifies the fundamentals of heat generation associated with FMR in nanomaterials; additionally, it not only opens up a new opportunity for the application of FMR to magnonics or magnetic hyperthermia, but also provides guidance for the design of advanced materials that enable highly localized heating with extra-high-energy power by choosing nanoparticles’ constituent materials of given saturation magnetization and damping parameters.

## Methods

### Micromagnetic simulation

We conducted finite-element micromagnetic (FEM) simulations at zero temperature on a single sphere of *M*Fe_2_O_4_ (*M* = Fe, Mn, Ni) and 2*R* = 12 nm diameter using the FEMME code (version 5.0.9)^41^, which incorporates the Landau-Lifshitz-Gilbert (LLG) equation. The integration of LLG ordinary differential equation (ODE) is achieved using the fourth-order Runge–Kutta methods as the predictor-correct solver. The curved surface of the model sphere was discretized into triangles of approximately equal area using Hierarchical Triangular Mesh^42^ (see Fig. 2a). The material parameters used in the simulations were as follows: magnetization at *H*_DC_ = 1 kOe, *M*_1kOe_ = 85.5 emu/g, Gilbert damping parameter *α* = 0.180, magnetocrystalline anisotropy *K*_1_ =  − 1.1 $\times 10^{5} \,{\text{erg/cm}}^{{3}}$ for Fe_3_O_4_, *M*_1kOe_ = 95.9 emu/g, *α* = 0.114, *K*_1_ =  − 0.3 $\times \;10^{5} \,{\text{erg/cm}}^{{3}}$ for MnFe_2_O_4_, and *M*_1kOe_ = 81 emu/g, *α* = 0.134, *K*_1_ =  − 0.62 $\times \;10^{5} \,{\text{erg/cm}}^{{3}}$ for the NiFe_2_O_4_ nanoparticle model. Due to the lack of experimental reports on exchange stiffness for MnFe_2_O_4_ and NiFe_2_O_4_, we set the *A*_ex_ value equal to $A_{{{\text{ex,Fe}}_{{3}} {\text{O}}_{{4}} }} = 13.2\;\;{\text{pJ/m}}$ for all of the sampled models. The values of *A*_ex_ and *K*_1_ were borrowed from other studies^43,44^, whereas the values of *M*_1kOe_ and *α* were directly obtained from VSM and VNA-FMR measurements, respectively (see Supplementary Information S3 for details). Thermal fluctuations at room temperature were not taken into account, because not only does the Zeeman energy at high DC fields of *H*_DC_ ~ 1kOe sufficiently exceed the thermal energy in the magnetic nanoparticles at room temperature, but also, *M*_S_ at room temperature is as sufficiently high, which is about 95, 75, and 90% of *M*_S_ at zero temperature for Fe_3_O_4_, MnFe_2_O_4_, and NiFe_2_O_4_, respectively^45^. Thus, our simulation results at *T* = 0 K can represent the general characteristics of the dynamic motions of magnetic nanoparticles. To excite resonant precession dynamic motions, we used CCW rotating fields ${\mathbf{H}}_{{{\text{rot}}}} = H_{{{\text{AC}}}} [\cos (2\pi f_{{{\text{AC}}}} t){\hat{\mathbf{x}}} + \sin (2\pi f_{{{\text{AC}}}} t){\hat{\mathbf{y}}}]$ for the simulation and linear oscillating fields ${\mathbf{H}}_{{{\text{lin}}}} = H_{{{\text{AC}}}} \sin (2\pi f_{{{\text{AC}}}} t){\hat{\mathbf{x}}}$ for the experiment, with *H*_AC_ and *f*_AC_ the amplitude and frequency of oscillating fields, respectively (See Supplementary Information S1 for different precession motions between **H**_lin_ and **H**_CCW_).

### Temperature measurements

Using a drop-casting method_,_ nanocrystalline particles were placed on the surface of a 400-μm-length Cu line in a microstrip. To excite magnetization dynamics in the particles, AC currents of different GHz frequencies using a signal generator (E8257D, Agilent) were applied to the microstrip and were amplified to several watts by a radio-frequency (RF) power amplifier (5170FT, Ophir) to generate sufficient strengths of AC magnetic fields (*H*_AC_) around the signal line. The magnetic field strength generated by AC currents flowing along the microstrip was calculated to be *H*_AC_ ~ 1.0 Oe with input power of 1 W^20^. By sweeping *H*_DC_ from 0 to 2.1 kOe at a rate of 7 Oe/s for each of *f*_AC_ = 1.5, 2.0. 2.5, and 3.0 GHz, we measured temperature increments using an infrared camera (T650sc, FLIR) to an accuracy of about ± 1 K at a maximum temporal resolution of 30 Hz. Temperature calibration was made by measuring boiling water (99.2 ± 2.1 °C) and melting ice (− 1.2 ± 0.2 °C) temperatures from the infrared emissivity of 0.98 and 0.97, respectively. Particle temperature was estimated by averaging the temperatures within a local 200 μm × 200 μm area at the center position of the signal trace.

## Supplementary Information

Supplementary Information.
