# Supplementary material for: Highly efficient heat-dissipation power driven by ferromagnetic resonance in MFe2O4 (M = Fe, Mn, Ni) ferrite nanoparticles
Source: Sci Rep. 2022 Mar 28;12:5232. doi: 10.1038/s41598-022-09159-z (PMC8960867; doi:10.1038/s41598-022-09159-z)
Supplement: Supplementary file 1 — Supplementary Information. [file 41598_2022_9159_MOESM1_ESM.docx]

**Supplementary Information**

**Highly efficient heat-dissipation power driven by ferromagnetic resonance in *M*Fe_2_O_4_ (*M* = Fe, Mn, Ni) ferrite nanoparticles**

Jae-Hyeok Lee, Yongsub Kim, and Sang-Koog Kim^*^

*National Creative Research Initiative Center for Spin Dynamics and Spin-Wave Devices, Nanospinics Laboratory, Research Institute of Advanced Materials, Department of Materials Science and Engineering, Seoul National University, Seoul 151-744, South Korea*

Supplementary Section 1 | Micromagnetic Simulation

Supplementary Section 2 | Synthesis of *M*Fe_2_O_4_ (*M* = Fe, Mn, Ni) nanoparticles and structural/magnetic properties

Supplementary Section 3 | Ferromagnetic resonance (FMR) measurements

Supplementary Section 4 | Estimation of resonance frequency *f*_res_ and damping parameter *α*

Supplementary Section 5 | Measurement of temperature incrementation

Supplementary Section 6 | Numerical calculation of *Q* versus time for different values of *H*_DC_ and *H*_AC_ for *M*Fe_2_O_4_ (*M* = Fe, Mn, Ni) materials

Supplementary Section 7 | Curves of temperature incrementation of nanoparticles versus *H*_DC_

according to different applications of *f*_AC_ and *H*_AC_

Supplementary Section 8 | Comparison of $\bar{\text{Q}}$_res_ versus power loss *P*_diss_

**S1. Micromagnetic Simulation**

**A. Numerical calculation of energy-dissipation rate *Q***

We performed a numerical calculation of energy-dissipation rate (power loss) *Q* from the micromagnetic simulation data. We applied different oscillating magnetic fields of a linearly oscillating AC field $\mathbf{H}_{\text{lin}}=H_{\text{AC}}\sin(2\pi f_{\text{AC}}t)\hat{\mathbf{x}}$, along with counter-clockwise (CCW) and clockwise (CW) circularly rotating fields $\mathbf{H}_{\text{CCW}}=H_{\text{AC}}\cos(2\pi f_{\text{AC}}t)\hat{\mathbf{x}}+H_{\text{AC}}\sin(2\pi f_{\text{AC}}t)\hat{\mathbf{y}}$ and $\mathbf{H}_{\text{CW}}=-H_{\text{AC}}\cos(2\pi f_{\text{AC}}t)\hat{\mathbf{x}}+H_{\text{AC}}\sin(2\pi f_{\text{AC}}t)\hat{\mathbf{y}}$, where *H*_AC_ and *f*_AC_ are the amplitude and frequency of the oscillating fields, respectively. Note that linearly oscillating magnetic fields can be composed of CCW and CW rotating fields of the same field amplitude and frequency, as $\mathbf{H}_{\text{lin}}=(1/2)(\mathbf{H}_{\text{CCW}}+\mathbf{H}_{\text{CW}})$. Figure S1 compares the temporal variations of *Q* for the model sphere of MnFe_2_O_4_ illustrated in Fig. 2(c), which was excited under a resonance field condition (*f*_AC_ = *f*_res_ = 3.0 GHz, *H*_DC_ = 1010 Oe) with *H*_AC_ = 3.0 Oe by either **H**_lin_ (blue line), **H**_CCW_ (red line), or **H**_CW_ (black line). For application of **H**_lin_, *Q* was plotted with the average value over a time period *T*_0_ (= 1/*f*_AC_) to obtain its measurable quantity^1^. Since the precession of magnetizations is CCW in its rotational sense, the CCW rotating field is the resonance field basis. Thus the application of **H**_CW_ does not contribute to the precession motion. Thus too, the application of **H**_lin_ contributes to the precession motion by one-fourth the effect of the **H**_CCW_ application, as shown in Fig. S1. We also notice that *Q* is affected by $H_{\text{AC}}^{2}$, as shown in Eq. (1). The steady-state energy-dissipation rate was estimated to be $\bar{Q}_{\text{res}}$= 11.6, 2.95, and ~ 0 kW/g for the application of **H**_CCW_, **H**_lin_, and **H**_CW_, respectively. Therefore, our experimental measurement of temperature incrementation by linear oscillating fields are comparable to the *Q* values calculated from the numerical data of the simulations carried out by CCW rotating fields. Only the difference of using the CCW rotating and linear oscillating fields leads to $Q_{\text{lin}}=Q_{\text{CCW}}/4$, as shown in Fig. S1.


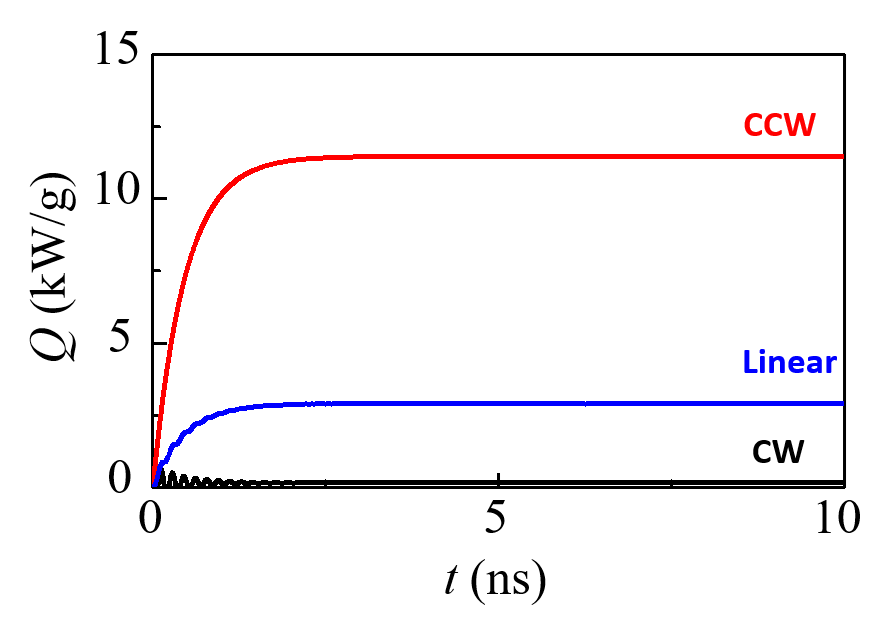


Figure S1. Energy-dissipation rate *Q* versus time for CCW (red line), CW (black line) rotating, and linearly (blue line) oscillating magnetic fields under resonance field condition (*f*_AC_ = 3.0 GHz, *H*_DC_ = 1010 Oe) with *H*_AC_ = 3.0 Oe for model sphere of MnFe_2_O_4_ material and 12 nm diameter.

**B. Calculation of *P*_Gibbs_, *P*_dual_, and *Q* = *P*_Gibbs_ + *P*_dual_ from simulation data for different *M*Fe_2_O_4_ materials (*M* = Fe, Mn, Ni)**

**
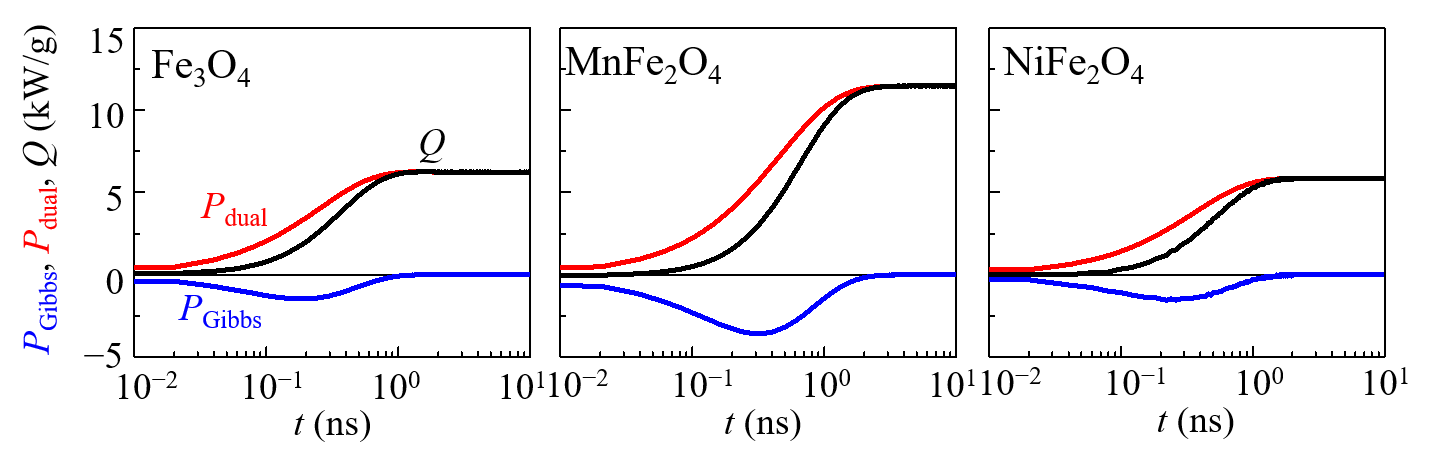
**

Figure S2. Energy-dissipation rate *Q* (black), dual power density *P*_dual_ (red), and total Gibbs free energy rate *P*_Gibbs_ (blue) calculated from numerical data of simulations excited by CCW rotating magnetic field under resonance field condition (*f*_AC_ = 3.0 GHz) with *H*_AC_ = 3.0 Oe for different materials Fe_3_O_4_ (left panel), MnFe_2_O_4_ (middle panel), and NiFe_2_O_4_ (right panel). To meet the resonance field condition for *f*_AC_ = 3.0 GHz, we used different values of *H*_DC_ = 970, 1010, and 980 Oe for the Fe_3_O_4_, MnFe_2_O_4_, and NiFe_2_O_4_ materials, respectively.

**S2. Synthesis of *M*Fe_2_O_4_ (*M* = Fe, Mn, Ni) nanoparticles and structural/magnetic properties**

**A. Synthesis:** Fe_3_O_4_ nanoparticles were synthesized through thermal decomposition of iron precursors in a hot organic solvent using a previously developed approach reported in Refs.[2-4]. Iron(II) chloride (3.25 mmol), iron(III) acetylacetonate (5.00 mmol), oleylamine (91.2 mmol), oleic acid (31.6 mmol) and octyl ether (49.9 mmol) were dissolved under an argon atmosphere. For the synthesis of MnFe_2_O_4_ and NiFe_2_O_4_, manganese chloride (MnCl_2_­) and nickel chloride (NiCl_2_) were added instead of iron chloride, respectively. The resulting mixtures were heated to 300 ^o^C and then refluxed for an hour. After cooling to room temperature, black-colored precipitates were isolated by cleaning with an excess amount of ethanol and the subsequent centrifugation. In order to make individual nanoparticles covered with silica shells, the mixture solution containing the *M*Fe_2_O_4_ nanoparticles, Igepal CO-520 (800 mg), ammonium hydroxide (105 μl), cyclohexane (12 ml) and tetraethyl orthosilicate of 40 μl for the shell thickness of 12 nm was maintained at room temperature for 72 h. Silica-coated nanoparticles were centrifuged with an excess amount of ethanol, followed by purification using a MACS column (Miltenyi Biotec) and dispersion in water after carboxylate functionalization.

**B. Structural and magnetic properties:** The shape and diameter of *M*Fe_2_O_4_ nanoparticles were examined by measurement of bright-field transmission electron microscopy (TEM) images. Figure S3(a) compares the TEM images of the different material nanoparticles, which revealed highly monodispersed and spherically shaped particles of an average diameter of *d* = 12 nm, as well as separation of the individual nanoparticles by 12 nm thick silica shells. The silica-shell coating prevents the magnetic particles from agglomerating and leads to subsequent suppression of inter-dipolar and inter-exchange interactions between the individual particles. The room-temperature field-decreasing magnetization curves (Fig. S3(b)) of the *M*Fe_2_O_4_ nanoparticles prior to silica coating exhibits superparamagnetic characteristics of zero coercivity and zero remanence along with sufficient saturation magnetization values of *M*_S_ = 101, 114, and 90 emu/g for Fe_3_O_4_, MnFe_2_O_4_, and NiFe_2_O_4_, respectively, which were measured under a sufficiently high field of *H*_DC_ = 45 kOe. The *M*_S_ values are normalized by dividing with the mass of magnetic ions excluding oxygens and silica shells.


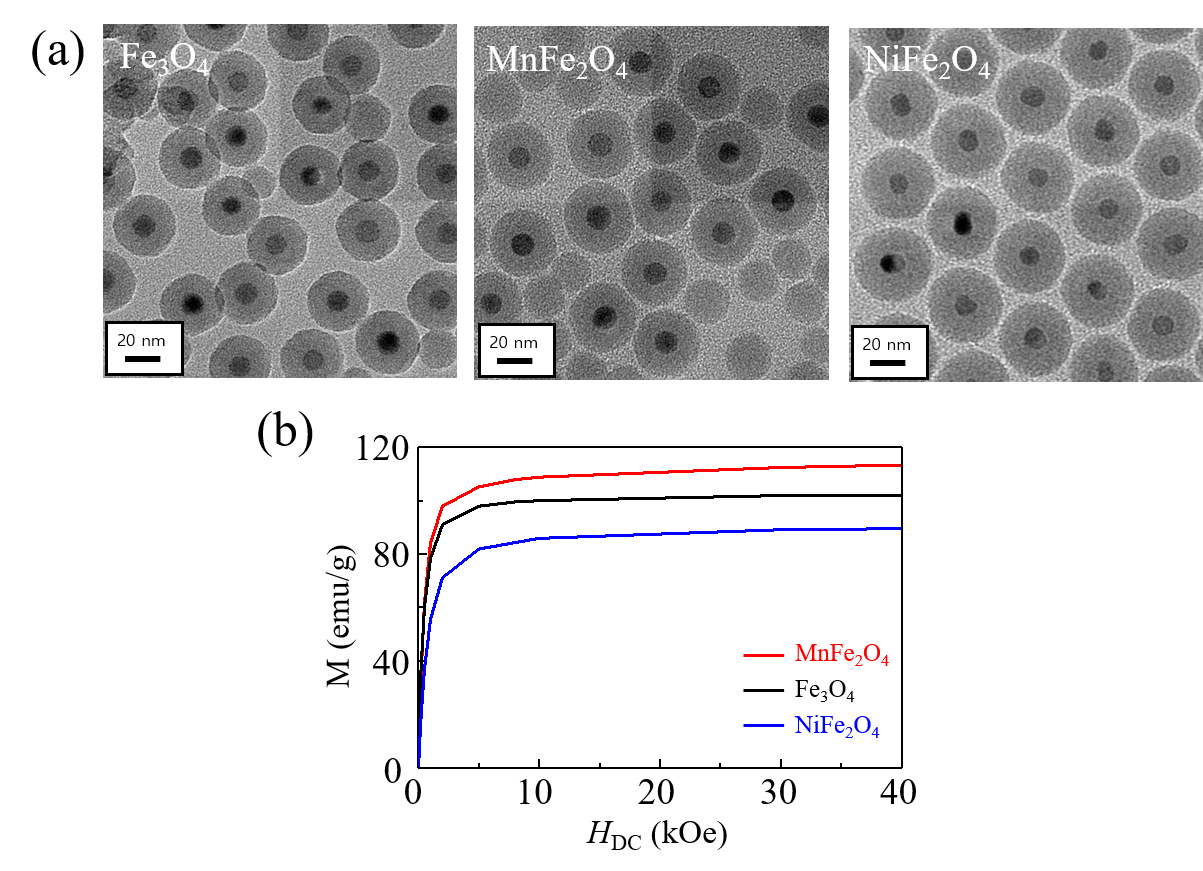


Figure S3. (a) Bright-field TEM images of *M*Fe_2_O_4_ (*M* = Fe, Mn, Ni) nanocrystals capped with 12 nm thick silica shells. (b) Room-temperature magnetization curves for *M*Fe_2_O_4_ nanocrystals prior to silica coating, as measured by VSM under decreasing magnetic field from 45 kOe to zero.

**S3. Ferromagnetic resonance (FMR) measurements**

To measure the precession of magnetizations in magnetic particles, we performed vector- network-analyzer-ferromagnetic-resonance (VNA-FMR) measurements^5^, as schematically shown in Fig. S4(a). The microstrip sample stage was connected to a VNA (E8362C, Agilent) with coaxial cables for high-frequency measurements and then placed on an electromagnet that applied DC magnetic fields parallel to the waveguide direction. We used non-magnetic End Launch SMA-type connectors to prevent interference with the high strengths of the DC magnetic fields. We measured the scattering parameter |*S*_21_| of the nanoparticle samples, where |*S*_21_| denotes the ratio of the measured powers at port 2 and port 1. Before the measurements, a standard short-open-load-through (SOLT) 2-port calibration procedure was performed to exclude systematic errors such as interference from cables, connectors, and/or the instrument itself. The measurement conditions were as follows: VNA frequency sweep, from 50 MHz to 10 GHz; intermediate frequency (IF), 500 Hz; the stimulus input power, 0 dBm. The strength of DC magnetic fields was varied from 0 to 2.4 kOe in stepwise increments of 20 Oe every 45 sec. The frequency-swept spectra under the given DC field strengths were obtained by the average of three separate measurements. The resultant |*S*_21_| spectra were finally obtained by subtracting the background signal under a sufficiently strong magnetic field of *H*_DC_ = 3.5 kOe, which field strength corresponds to non-resonant excitation.

The resultant transmission power spectra of |∆*S*_21_| =$\left|S_{21, H}-S _{21, H=3.5\text{kOe}}\right|$ for the three different nanoparticle materials are shown in Fig. S4(b). The contour plots of |∆*S*_21_| on the *f*-*H*_DC_ plane clearly exhibit typical FMR behavior of superparamagnetic nanoparticles: the resonant microwave absorption at a specific frequency of AC fields for a given value of *H*_DC_ represents a strong FMR effect of given material nanoparticles, and the precession frequency increases with the DC field strength, being well in agreement with Kittel’s equation^6^. The intensity of the absorbed transmission powers |∆*S*_21_| for the MnFe_2_O_4_ material was the largest among the materials.

**
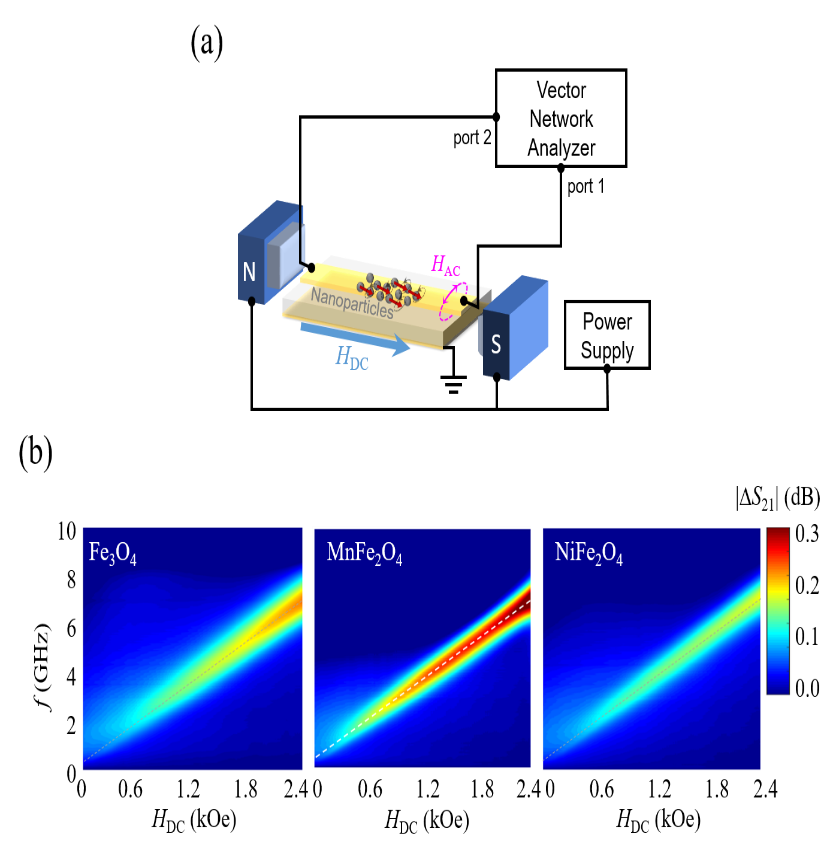
**Figure S4. (a) Schematic drawing of VNA-FMR set-up along with microstrip sample stage on which nanoparticles were placed. The directions of the applied DC and AC magnetic fields are indicated by the respective arrows. (b) |∆*S*_21_| spectra on *f*_AC_-*H*_DC_ plane for silica-shell-coated *M*Fe_2_O_4_ particles (*M* = Fe, Mn, Ni). The dotted lines represent linear fits to the experimental data using the Kittel equation.

**S4. Estimation of resonance frequency *f*_res_ and damping parameter *α***

For non-negligible damping, the resonance frequency of precession in a single-domain nanosphere can be expressed as^7^

$f_{\text{res}}=\gamma_{\text{G}}H_{\text{eff}}=\frac{\gamma_{0}}{1+\alpha^{2}}(H_{\text{DC}}+H_{\text{int}})$, (S1)

where *γ*_G_ and *γ*_0_ are the Gilbert gyromagnetic ratio and the electron gyromagnetic ratio, respectively, and *H*_int_ is the internal field. From fitting using Eq. (S1) to the experimental data shown in Fig. S4(b), *α* and *H*_int_ were estimated to be *α* = 0.180, 0.114, and 0.134, and $H_{\mathrm{int}}$= 174, 125, and 161 Oe for Fe_3_O_4_, MnFe_2_O_4_, and NiFe_2_O_4_, respectively, for a constant value of *γ*_0_/2π = 2.8 MHz/Oe, as summarized in Table S1. In the fitting, we used the data only for the region of *H*_DC_ > 1 kOe, because the magnetizations of the superparamagnetic nanoparticles were sufficiently aligned in the direction of *H*_DC_. The internal field originated from the magnetocrystalline anisotropy of the ferrimagnetic materials (*K*_1_ = −1.1, −0.3, and −0.62 $\times10^{5} \text{erg/cm}^{3}$ for M = Fe, Mn, and Ni, respectively^8^) whose field axes are randomly oriented, as well as from intra-dipolar interaction inside each particle due to the imperfection of the sphere shape and possible disorders on the particle surfaces. Inter-dipolar and inter-exchange interactions between neighboring magnetic nanoparticles can be induced by the agglomeration of magnetic particles^9^. But in our case, where the particles were separated by silica shells, the internal field *H*_int_ would be mostly caused by the magnetocrystalline anisotropy field as well as the intra-dipolar interaction of each particle.

Table S1. Material parameters of *M*Fe_2_O_4_ (*M* = Mn, Fe, Ni) nanoparticles estimated from experimentally measured FMR spectra

|  | Fe_3_O_4_ | MnFe_2_O_4_ | NiFe_2_O_4_ |
| --- | --- | --- | --- |
| (*γ*_G_/2π)[MHz/Oe] | 2.712±0.003 | 2.764±0.004 | 2.751±0.003 |
| *α* | 0.180±0.003 | 0.114±0.006 | 0.134±0.008 |
| *H*_int_ [Oe] | 174.4±1.9 | 124.8±2.3 | 160.7±1.4 |

**S5. Measurement of temperature incrementation**

The left column of Fig. S5 shows optical images of the nanoparticles placed on a microstrip sample stage. The middle and right columns show their IR images before and after the application of a resonance field (*f*_AC_ =3.0 GHz) with *H*_AC_ = 3 Oe for 1 sec duration. During the temperature measurements, DC magnetic fields of *H*_DC_ = 1030, 1014, and 1017 Oe were applied for Fe_3_O_4_, MnFe_2_O_4_, and NiFe_2_O_4_, respectively, in order to satisfy the corresponding resonance conditions with the frequency of *f*_AC_ =3.0 GHz. In the AC field-off IR images, the purple color in the middle corresponds to room temperature (*T*_room_ ~ 25℃) of the magnetic particles. At 1 sec immediately after applying the AC field, the color of the particles becomes yellow, as observed only in the region of the particles. Depending on the materials, the temperature increments were estimated contrastingly by Δ*T* ~ 8, 12, and 7 ℃ for Fe_3_O_4_, MnFe_2_O_4_, and NiFe_2_O_4_, respectively. These IR images clearly reveal that the temperature incrementation of nanoparticles can be quantitatively measured via thermal radiation during ferromagnetic resonance and its consequent dissipation process.


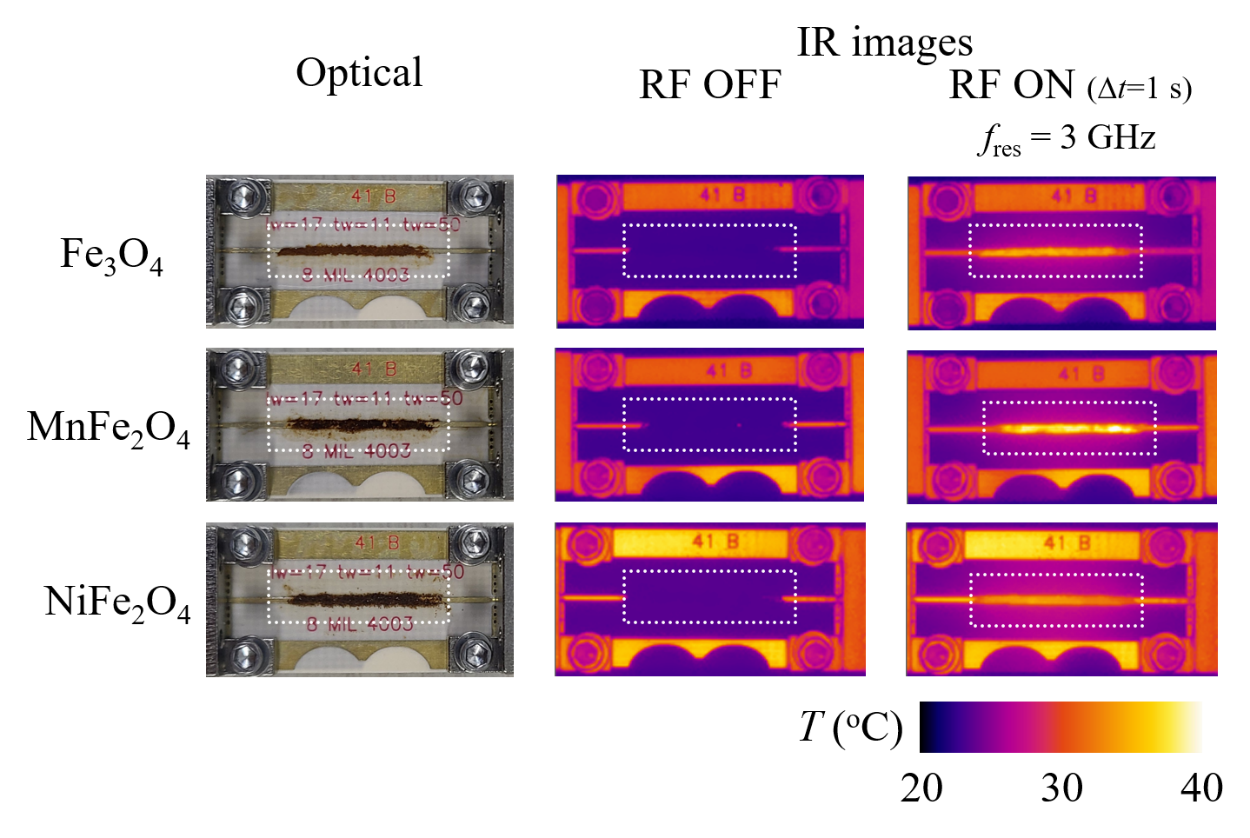


Figure S5. Optical images (left column) of *M*Fe_2_O_4_ (*M* = Fe, Mn, Ni) nanoparticles on sample stage and thermal IR images for AC field off (middle column) and at 1 sec immediately after application of resonance AC field (right column). The color bar on the bottom indicates the local temperatures.

**S6. Numerical calculation of *Q* versus time for different values of *H*_DC_ and *H*_AC_ for *M*Fe_2_O_4_ (*M*= Fe, Mn, Ni) materials**

**A. Energy-dissipation rate *Q* versus time for different field strengths of *H*_DC_**


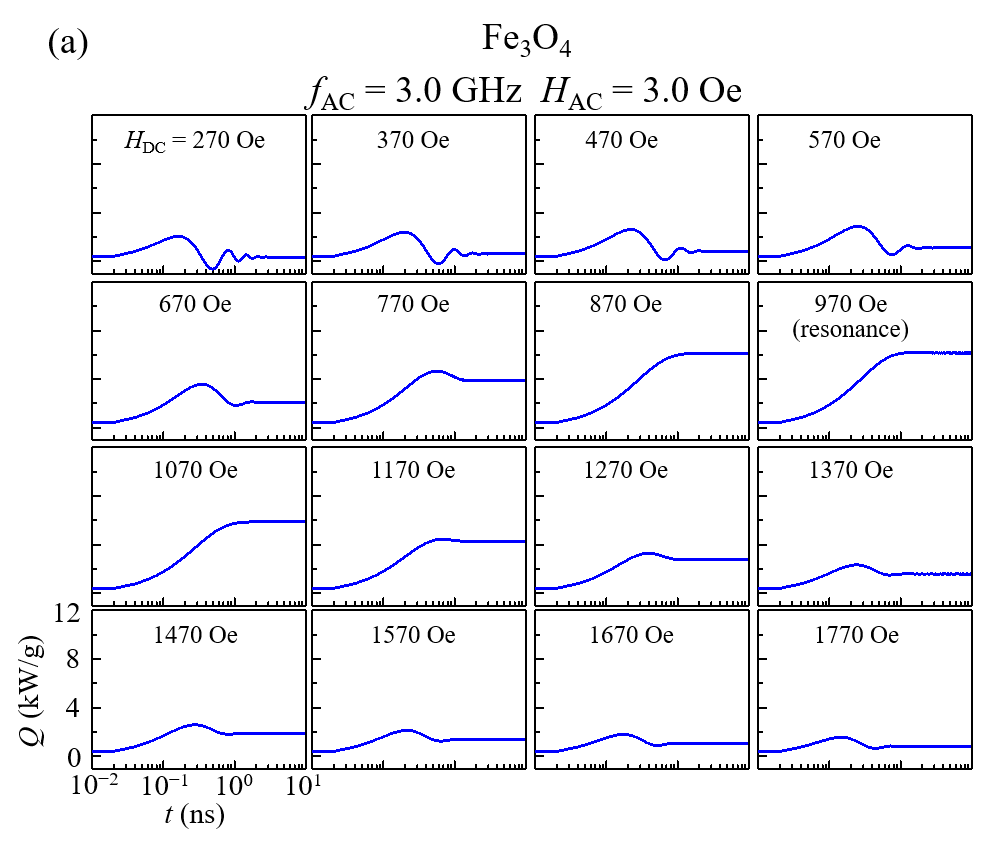


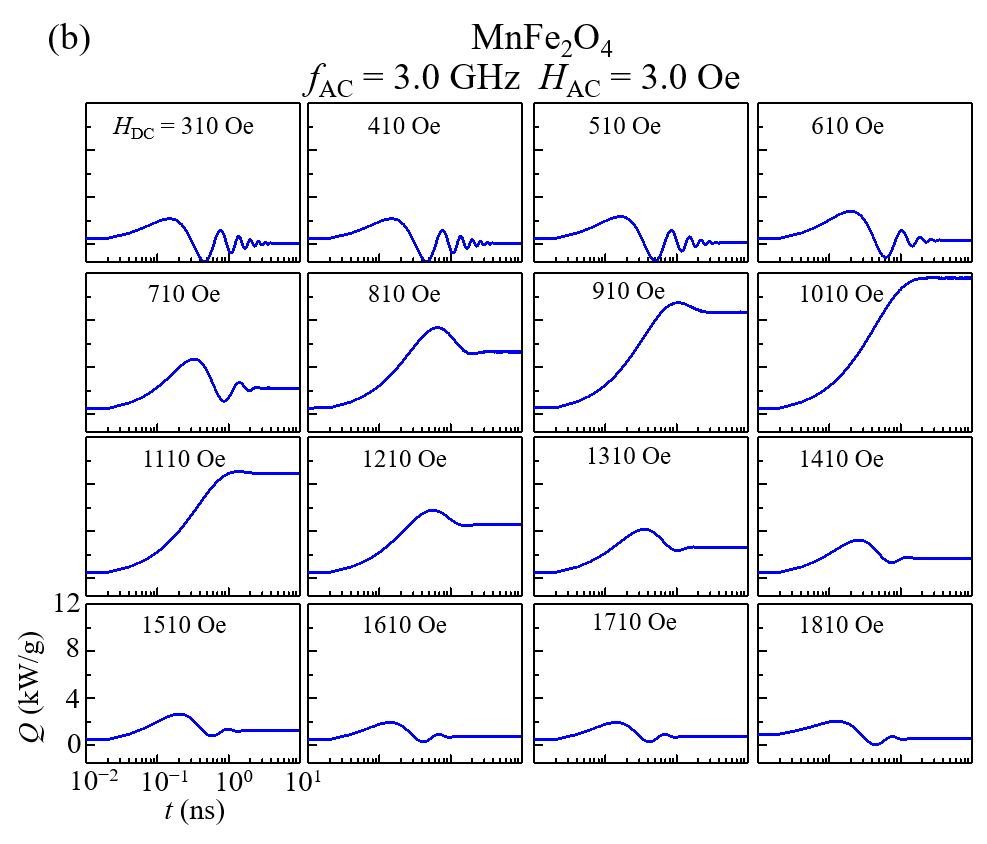


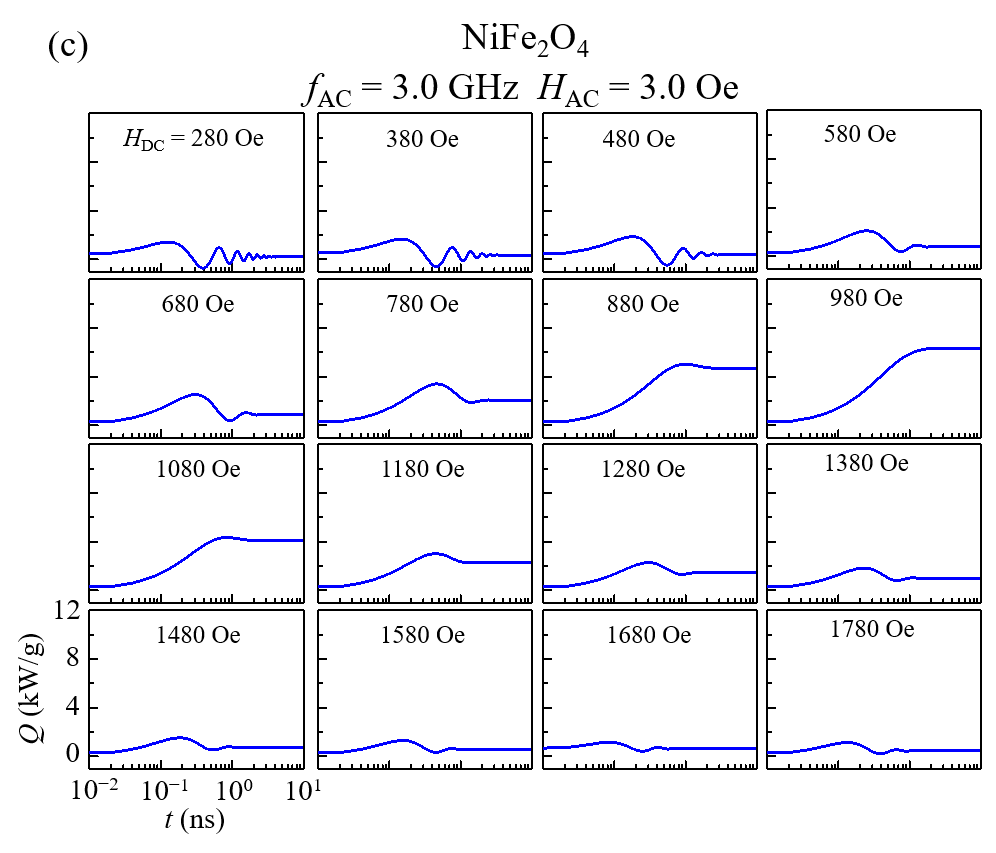


Figure S6(a-c). Energy-dissipation rate *Q* versus time as obtained by calculation of numerical data of micromagnetic simulations by CCW rotating field of *f*_AC_ = 3.0 GHz and *H*_AC_ = 3.0 Oe for indicated different strengths of DC magnetic fields for (a) Fe_3_O_4_, (b) MnFe_2_O_4_ , and (c) NiFe_2_O_4_ nanosphere models.

**B. Energy-dissipation rate *Q* versus time for different field strengths of *H*_AC_**


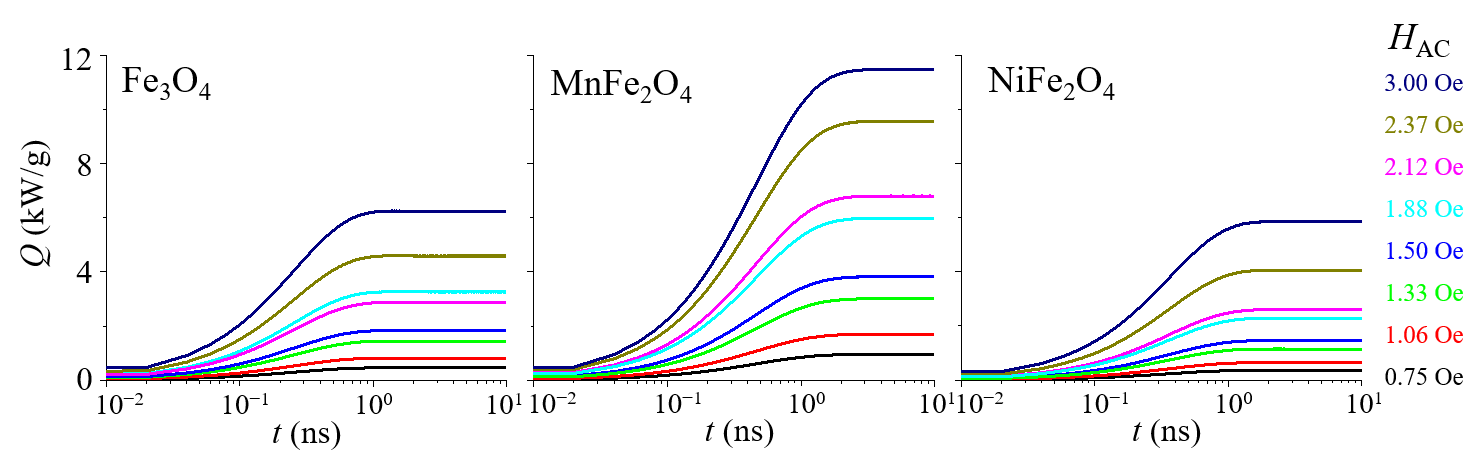


Figure S7. Energy-dissipation rate *Q* versus time as numerically calculated from simulation data for single-sphere models of Fe_3_O_4_, MnFe_2_O_4_, and NiFe_2_O_4_ for application of CCW rotating magnetic fields with different values of *H*_AC_ ranging from 0.75 to 3.00 Oe under resonance field condition of *f*_res_ = 3.0 GHz. Note that different DC field strengths *H*_DC_ = 970, 1010, and 980 Oe were applied for the Fe_3_O_4_, MnFe_2_O_4_, and NiFe_2_O_4_ materials, respectively, to meet the resonance field condition.

**S7. Curves of temperature incrementation of nanoparticles versus *H*_DC_ according to different applications of *f*_AC_ and *H*_AC_**

**
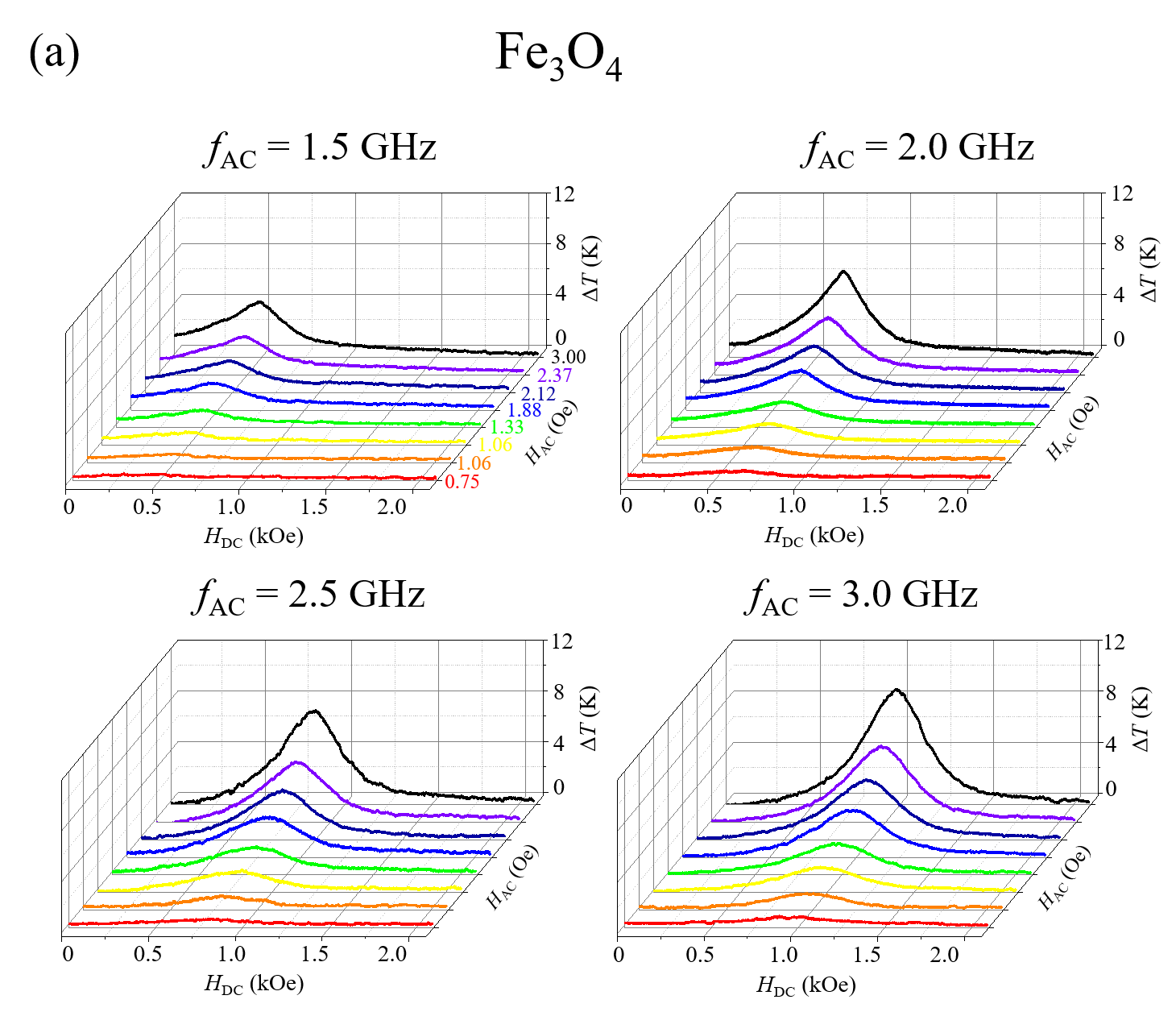
**

**
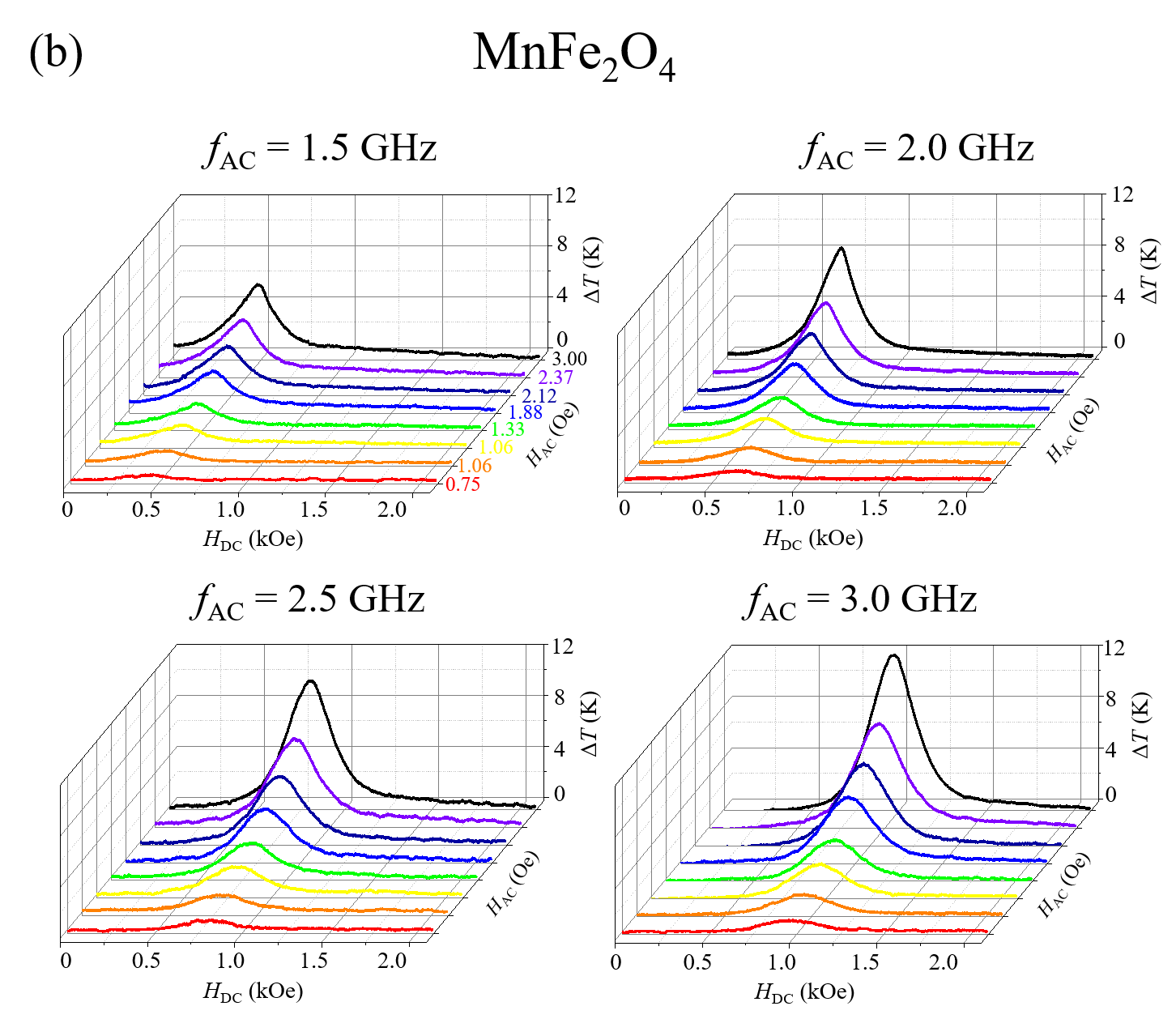
**


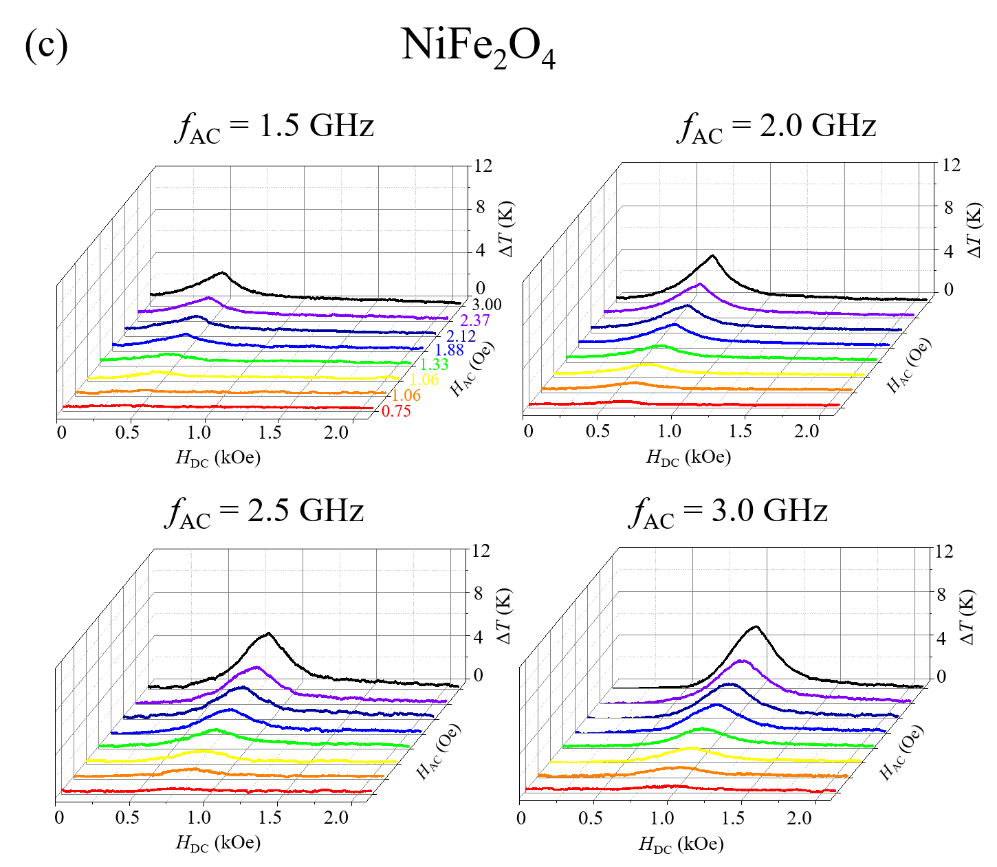
Figure S8(a-c). Experimentally measured temperature incrementation versus *H*_DC_ for different AC magnetic field strengths *H*_AC_ (*H*_AC_ = 0.75 ~ 3.00 Oe) for four different AC frequencies (*f*_AC_ = 1.5, 2.0, 2.5, and 3.0 GHz) for (a) Fe_3_O_4_, (b) MnFe_2_O_4_, and (c) NiFe_2_O_4_ nanoparticles

**S8. Comparison of $\bar{\text{Q}}$_res_** **versus power loss *P*_diss_**

We notice that the analytical form of $\bar{Q}_{\text{res}}$ shown in Eq. (1) is the same as the magnetic dynamic loss term^10^ $P_{\text{diss}}=\pi f_{\text{AC}}\mu_{0}\chi''H_{\text{AC}}^{2}$ with *χ"* the imaginary part of the dynamic susceptibility, *μ*_0_ the permeability of vacuum, and *H*_AC_ the linear oscillating field strength. For single-domain spherical-shape magnetic particles, *χ"* under the resonance condition can be expressed as^11^ $\chi''_{\text{FMR}}=\frac{\gamma\mu_{0}M_{S}}{4\pi f_{\text{res}}\alpha}$ with *f*_res_ the intrinsic resonance frequency of precession. Thus, *P*_diss_ at resonance is finally rewritten as $P_{\text{diss}}=\frac{\gamma\mu_{0}M_{S}}{4\alpha}H_{\text{AC}}^{2}$, which becomes exactly the same as $\bar{Q}_{\text{res}}\cdot\rho$ with *ρ* the density of magnetic material. Note that there is a difference in the scaling factor 1/4 due to the applications of the different oscillating fields (**H**_lin_ for *P*_diss_ and **H**_rot_ for $\bar{Q}_{\text{res}}$, as previously discussed in Supplementary Section S1). Accordingly, the steady-state energy-dissipation rate $\bar{Q}_{\text{res}}$ studied here exhibits the same quantity as the power loss caused by magnetization dynamic dissipation under the resonance condition, as reported in Ref. [11].

**REFERENCES**

1. M.-K. Kim *et al.*, Dynamical Origin of Highly Efficient Energy Dissipation in Soft Magnetic Nanoparticles for Magnetic Hyperthermia Applications. *Phys Rev Appl* **2018,** *9* (5).

2. J. Cheon, J.-W. Lee & J.-H Lee, Preparation method of magnetic and metal oxide nanoparticles. U.S. Patent No. 8,066,969. 29 Nov **2011**; J.-H. Lee et al., Artificially engineered magnetic nanoparticles for ultra-sensitive molecular imaging. *Nat Med* **2007,** *13* (1), 95-99.

3. J.-H. Lee et al., Exchange-Coupled Magnetic Nanoparticles for Efficient Heat Induction, Nature nanotechnology 2011, 6, 418-422

4. H. C. Davis *et al.*, Nanoscale Heat Transfer from Magnetic Nanoparticles and Ferritin in an Alternating Magnetic Field. *Biophys. J*. **2020**, 118, 1502–1510.

5. B. Kim *et al.,* Hetero-interface effect on Gilbert damping in nonmagnetic metal/permalloy/nonmagnetic metal trilayers. *J. Magn. Magn. Mater.* **2018,** *465*, 399-405.

6. C. Kittel, On the Theory of Ferromagnetic Resonance Absorption. *Phys. Rev.* **1948,** *73* (2), 155-161.

7. I. S. Poperechny, Y. L. Raikher, Ferromagnetic resonance in uniaxial superparamagnetic particles. *Phys. Rev. B* **2016,** *93* (1).

8. B.D. Cullity, *Introduction to magnetic materials*. Addison-Wesley Pub. Co.: Reading, Mass., **1972**; p xvii, 666 p.

9. E. de Biasi *et al.*, Size and anisotropy determination by ferromagnetic resonance in dispersed magnetic nanoparticle systems. *J. Magn. Magn. Mater.* **2003,** *262* (2), 235-241.

10. L. D. Landau *et al.*, *Electrodynamics of continuous media*. 2nd ed.; Pergamon: Oxford Oxfordshire ; New York, **1984**; p xiii, 460 p.

11. B. Lax, K. J. Button, *Microwave Ferrites and Ferrimagnetics*. McGraw-Hill: **1962**.
